# Exertional Dyspnea as the Main Symptom in an Adolescent Athlete With Coronary Artery Anomaly – A Case Report

**DOI:** 10.3389/fcvm.2022.872608

**Published:** 2022-04-11

**Authors:** Mahdi Sareban, Klaus Hergan, Peter Covi, Josef Niebauer

**Affiliations:** ^1^University Institute of Sports Medicine, Prevention and Rehabilitation and Research Institute of Molecular Sports Medicine and Rehabilitation, University Hospital Salzburg, Paracelsus Medical University, Salzburg, Austria; ^2^University Institute of Radiology, University Hospital Salzburg, Paracelsus Medical University, Salzburg, Austria; ^3^Department of Pediatrics, University Hospital Salzburg, Paracelsus Medical University, Salzburg, Austria

**Keywords:** echocardiography, exercise testing, unroofing, arrhythmias, syncope, pre-participation examination, competitive sports

## Abstract

Coronary artery anomalies (CAA) are associated with sudden cardiac death (SCD) and the majority of those events occur during exercise. Depending on the anatomic features and severity, CAA usually provoke clinical symptoms of coronary ischemia, mainly syncope and (exertional) chest pain. Here we present a case of a female adolescent athlete with a high-risk CAA variant and an unusual clinical presentation, which delayed diagnosis 2 years after first symptoms were reported. After successful surgical management of the anomalous artery, the patient was determined eligible for competitive sports with unremarkable follow-up examinations.

## Introduction

Coronary artery anomalies (CAA) are congenital disorders defined as an abnormal origin or course of any or the three main epicardial coronary arteries and is estimated to affect 0.2–5.8% of the population, based on criteria and the diagnostic method used (1). CAA are associated with sudden cardiac death (SCD) and the majority of those events occur during exercise (2). In a recent prospective surveillance investigating the etiology and incidence of SCD in US competitive middle school, high school, and college athletes, CAA were responsible for 12.0% of all events. Notably, CAA were the most common cause of SCD in middle school athletes (28%) but represented only 12% of cases in high school athletes and 3% of cases in college and professional athletes (3). Here we present a case of an adolescent athlete with a high-risk CAA variant and an unusual clinical presentation.

## Case Description

**FIGURE 1:**
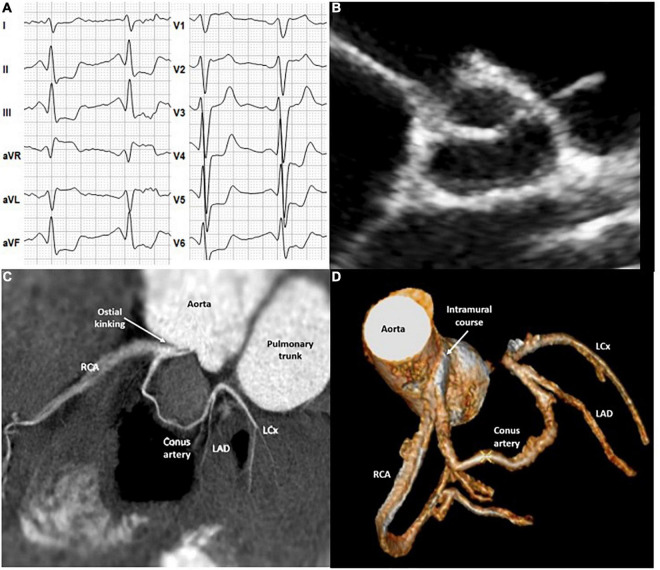
**(A)** Exercise electrocardiogram. **(B)** Transthoracic echocardiography. **(C,D)** Coronary computed tomography angiography. LCx, Left circumflex artery; LAD, Left anterior descending artery; RCA, Right coronary artery.

A 16-year old female gymnast (height: 150 cm; body mass: 42 kg) was referred to our institute due to progressive exertional dyspnea and exercise intolerance during the previous 2 years without established diagnosis. Medical history revealed an exercise-related syncope 2 weeks prior to referral. Her family history was unremarkable. Resting and exercise ECGs performed 2 years prior to the current examinations were without significant abnormalities. Current resting ECG demonstrated marginal (1 mm) horizontal ST-segment depression in leads II, III, and aVF. Exercise testing revealed reduced maximal exercise capacity (105 W; 2.5 W/kg), inadequate chronotropic response and significant (5 mm) horizontal ST-segment depression in leads II, III, aVF, and V4-V6 (Figure 1A). Upon transthoracic echocardiography (TTE), no coronary artery ostia could be displayed at the level of the aortic valve (Figure 1B). Coronary computed tomographic angiography (CTA) demonstrated an atresia of the left main coronary artery, a single ostium with high takeoff at the ascending aorta above the left sinus of Valsalva with a stenosis due to ostial kinking (Figure 1C: CTA) and an intramural course within the aortic wall (Figure 1D: 3D CTA reconstruction). A thin conus artery branched off two cm after the common trunk and a left anterior descending and a thin circumflex artery from the conus artery whereas a large right coronary artery could be displayed until the periphery (Figure 1D: 3D CTA reconstruction). The patient was managed surgically with unroofing of the intramural segment of the anomalous artery, that is, an incision of the length of the intramural portion and a formation of a neo-orifice (4). Six months after surgery the patient was free of symptoms and exercise testing revealed improved maximal exercise capacity [176 W; 4.1 W/kg)], regular chronotropic response and complete resolution of previous ST-segment depression, so that the patient was determined eligible for competitive sports. Twenty months after surgery exercise capacity further increased to 214 W (4.5 W/kg) without clinical or electrocardiographic signs of myocardial ischemia.

## Discussion

Cardiovascular disease is the leading cause of sudden death, also in adolescent athletes (5). Since it is generally assumed that athletes are in great health, as in our case, symptoms commonly do not receive appropriate responses and consequently diagnosis of underlying cardiac disease is often delayed. CAA is the second most common cause of SCD in young adolescent athletes (3) and also present a risk factor for SCD in a non-athletic population (6).

Depending on the anatomic features and severity, CAA usually provoke clinical symptoms of coronary ischemia secondary due to ostial kinking and/or an intramural course of the anomalous vessel and/or compression of the anomalous vessel between the aorta and the pulmonary artery (i.e., interarterial course) (7). In a review of registries of young competitive athletes with autopsy proven CAA as the sole cause of SCD and available clinical data before SCD occurred, syncope and exertional chest pain were the most common symptoms (2). This is in line with another registry where common symptoms included syncope, (exertional) chest pain, and resuscitated SCD. However, most of these patients hat no symptoms and the diagnosis was established within the diagnostic work-up of an incidental heart murmur (8).

Furthermore, neither routine resting 12-lead- nor exercise electrocardiograms (ECG) yield high diagnostic value, especially in young adolescents (2). This is mirrored in our case, since the patient had unremarkable resting- and exercise ECGs when she first reported symptoms but demonstrated significant abnormalities after 2 years when the final diagnosis was established. Current guidelines on the management of adults with congenital heart disease suggest the use of a stress test either when CAA are an incidental finding or when their clinical significance cannot be completely assessed from anatomic studies (9). Notably, no standardized stress protocols have been proposed so far to stratify CAA-induced ischemia but exercise ECG is often the first test performed considering its cost-effectiveness and wide availability (10). This case also highlights the value of TTE for the prevention of exercise-related SCD, even in case of an asymptomatic athlete with unremarkable resting- and exercise ECG. In Europe, 65% of physicians involved in pre-participation screening of athletes use TTE as part of their standard protocol, even in the setting of a normal clinical evaluation and resting ECG (11). Of note, in a study with short (5 min) TTE protocol tailored for pre-participation screening of athletes, the origin of the left coronary artery was identified in 99% and the origin of the right coronary artery in 96% of the cases (12). Thus, displaying the ostia of the coronaries should be part of the standard TTE protocol not only when screening athletes but is obligatory whenever syncope or exertional symptoms including dyspnea are reported. In the present case, the orifices of the coronary arteries could not be visualized at the sinus of aorta upon TTE due to a high takeoff of the single coronary artery from the ascending aorta. Thus, TTE was non-diagnostic or inconclusive in this case, prompting further imaging. CTA is currently considered the gold standard for establishing the diagnosis and assessing the risk for SCD, especially in case of persisting symptoms but other investigations are inconclusive. CTA provides a more detailed assessment of coronary artery anatomy with cardiac magnetic resonance being an alternative. Both examinations yield very high negative predictive value and thus may rule out CAA, but are also relevant for considering differential diagnosis, such as atherosclerotic obstructive coronary artery disease and myocardial bridging. Of note, myocardial bridging present an intramural tract as seen in this case, but has normal coronary artery origin. Considering the widespread availability of CTAs, coronary catheter-based angiography is rarely used in the diagnostic work-up in adolescents with ischemia-like symptoms due to its invasive nature. Another differential diagnosis to consider are coronary artery spasm, where invasive coronary angiography may be considered to establish the diagnosis by intracoronary acetylcholine-testing. However, in intracoronary spasms thoracic pain usually presents at rest or during ordinary activity and are usually found in patients with underlying atherosclerotic coronary arteries (13).

The prognostic consequences and subsequently management of CAA are extremely variable, and each therapeutic choice should be tailored to the patient’s characteristics and the choice of surgical technique depends mostly on the origin of the anomalous vessel and the extent of the intramural tract (4). Anomalous aortic origin of a coronary artery is frequently characterized by a stenosis due to ostial kinking and an intramural course within the aortic wall, which can be safely repaired by unroofing the intramural segment, as performed in this study (4, 9).

Eligibility for sport competition participation needs to take coronary anatomy and the presence of inducible ischemia into account. High risk anatomy as indicated by orifice >1 cm above the sinotubular junction, stenosis due to ostial kinking, and the intramural course as seen in this case are established exercise-related SCD risk factors, since vigorous systolic expansion of the aorta during exercise may lead to further coronary artery kinking and occlusion. Thus, signs of ischemia within exercise stress testing preclude participation in most competitive sports with a moderate and high cardiovascular demand (14). In asymptomatic individuals with an anomalous coronary artery that does not course between the large vessels or does not have a ostial kinking with reduced lumen or intramural course, participation in competitive sports may be considered after adequate counseling on the risks provided that there is an absence of inducible ischemia (14). In case of successful surgical repair of CAA, return to all sports may be considered 3 months after surgery in asymptomatic athletes in the absence of inducible myocardial ischemia or complex cardiac arrhythmias during maximal exercise testing (14). Cardiopulmonary exercise testing enables readmitting a patient to exercise practice following surgical repair based on individual functional capacity.

In the present case, 6 months after surgery the patient was free of symptoms and exercise testing revealed complete resolution of previous ST-segment depression, so that the patient was determined eligible for competitive sports with improvement of exercise capacity from 105 Watt (2.5 W/kg) to 214 W (4.5 W/kg) without clinical or electrocardiographic signs of myocardial ischemia 20 months after surgery.

## Data Availability Statement

The original contributions presented in the study are included in the article, further inquiries can be directed to the corresponding author.

## Ethics Statement

Ethical review and approval was not required for the study on human participants in accordance with the local legislation and institutional requirements. The patient provided written informed consent to participate in this study.

## Author Contributions

MS wrote the first draft of the manuscript. KH contributed to the radiologic discussion of the manuscript and provided CT images for the manuscript. PC and JN contributed to the (differential) diagnostic discussion of the manuscript. All authors contributed to the article and approved the submitted version.

## Conflict of Interest

The authors declare that the research was conducted in the absence of any commercial or financial relationships that could be construed as a potential conflict of interest.

## Publisher’s Note

All claims expressed in this article are solely those of the authors and do not necessarily represent those of their affiliated organizations, or those of the publisher, the editors and the reviewers. Any product that may be evaluated in this article, or claim that may be made by its manufacturer, is not guaranteed or endorsed by the publisher.
